# Vida PURA: feasibility of culturally adapted screening and brief intervention for Latino day laborers

**DOI:** 10.1186/1940-0640-10-S1-A45

**Published:** 2015-02-20

**Authors:** India J Ornelas, Emily Williams, Bonnie Duran, Dennis Donovan

**Affiliations:** 1 Department of Health Services, University of Washington, Seattle, WA, 98195, USA; 2 Department of Health Services, Health Services Research & Development, Veterans Affairs Puget Sound Health, Seattle, WA, 98195, USA; 3 School of Social Work, University of Washington, Seattle, WA, 98195, USA; 4 Department of Psychiatry and Behavioral Sciences, University of Washington, Seattle, WA, 98195, USA

## Aim

Research suggests that Latino immigrant men face difficulties in adapting to life in the United States. One of the ways Latino men cope with these stressors is with heavy alcohol use. The aim of this study was to assess the feasibility of culturally adapted screening and brief intervention (SBI) to reduce heavy alcohol use in this population.

## Methods

We conducted qualitative interviews with Latino day laborers and social services to inform the cultural adaptation of screening and brief interventions. Interviews and focus groups were conducted by trained bilingual research staff. Recordings were transcribed; then transcripts were coded and analyzed in Atlas.ti. Each transcript was coded by two members of the research staff. Case summaries and coded quotations were reviewed for prevalent themes. Themes were used to identify sources of mismatch between traditional screening and brief intervention (SBI) and the target population. The adapted intervention was then pilot-tested to assess the feasibility and potential effectiveness. In the pilot test, 104 men were screened using the AUDIT, and men with a score ≥ 6 were offered a brief intervention (56%). Those receiving an intervention completed follow-up surveys at 2 and 8 eight weeks. Alcohol use was assessed using the AUDIT and 14 day timeline follow-back.

## Results

Findings from the qualitative interviews indicated that unhealthy drinking was common among Latino day laborers. Their drinking was related to and helped relieve immigration-related stressors. Men preferred to receive information from trusted providers in Spanish. They faced many barriers to accessing health and social services, and few culturally appropriate alcohol-related services existed. Based on these findings, we adapted SBI to incorporate the social and cultural context of Latino day laborers. SBI was provided in a community setting (at a day-labor worker center) by bilingual community health workers. Men were receptive to SBI during the pilot test. Results from the pilot test confirmed that unhealthy alcohol use was prevalent (average of 8.5 drinks per drinking day and 4 drinking days in past 14 days among intervention group). We were able reach 62 percent of the men at the 2 week and 57 percent at 8 week follow-up. Mean AUDIT scores among those receiving the intervention went from 18.7 at baseline, to 13.5 at 2 weeks, and 14.8 at 8 weeks.

## Conclusions

Our results suggest that Latino immigrant men have patterns of unhealthy alcohol use and are an underserved population. Evidenced-based interventions conducted in clinical settings, such as screening and brief intervention, may be more efficacious for Latino day laborers if conducted by community health workers in community settings where men more frequently seek services. Our findings can be used to further test culturally adapted SBI to prevent and reduce unhealthy alcohol use in this vulnerable population.

